# Women in Industry

**Published:** 1919-08-16

**Authors:** 


### WOMEN IN INDUSTRY.

### A Review of Cause and Effect.

In the guise of a review of the Report of the War Cabinet Committee on Women in Industry, the *Quarterly Review* for July has an important article on this subject by Sir Lynden Macassey, who was a member of that committee, and who has been engaged from the beginning of the war in organising women's labour.

The need of increasing production is now an accepted fact, but the "latent and unutilised capacities for production possessed by women" are unrecognised as yet. The article explains the problem from the economic point of view, and is divided into the three parts—before the war, during the war, and for the future.

### Before the War.

"One may accurately summarise the pre-war position of women in industry by saying that it was pre-eminently characterised by a demarcation of work which handicapped severely women's potentialities, and by conditions of work which seriously impaired their physical and mental well-being." Such demarcation is economically sound so far as it excluded women from work beyond the compass of their strength, but only a comparatively small amount of it can be so explained. For the rest, it was due to the success of the men and their trades unions in claiming all the best and most highly remunerated classes of work, and to the prejudice of many employers to women workers. The conditions of work are described as "dehumanising." Wages were pittances, owing to the supply of women at such rates being ample. Many worked merely for pocket-money, others as a supplement to the family wage; they were unorganised, and had no power of collective bargaining. As a result woman labour was regarded as "cheap" or of the "blackleg" type. Environment was inferior to that of the men, owing to want of trades-union pressure and to shortage of factory inspectors.

### During the War.

"At the end of the war, as compared with pre-war days, women had literally leapt, as agents of production, and by inherent economic powers and aptitude, into a position of eminence in the industrial work previously undreamt of even by themselves." Many had replaced men, and the "character of the work in 'women' trades was fundamentally altered." In skilled and jobbing work they were less efficient, but then the whole war period was less than the normal time of apprenticeship, and in comparison with youths put on at the same time they learnt as quickly or quicker. On work involving severe physical effort they did less in a given time than men. In "excess-production" work they proved equal, often superior, to men. "They seemed temperamentally immune to the deadening effect of monotonous work, to which men, on the other hand, are peculiarly susceptible." On the other hand, in work which needed a "combination of quick intelligence and manual dexterity within a limited ambit, women were invariably superior to men."

The statistical information upon which these general conclusions have been based is not disclosed in detail; possibly the summing up of the evidence is based substantially upon the opinions of those best qualified to testify as to conditions prevailing within specific industries. An estimate of the quality of the women worker when compared with the man worker may theoretically be improved when we reflect that the war woman worker was often drawn from scratch material, whereas the "indispensable" male worker, a trained person, was very largely in his place throughout the war.

### The Future.

Sir Lynden lays down three master principles which should regulate women's future station in industry. The paragraph is worth quoting in full:

"First, women should always be entitled to such employment as is fully commensurate with their economic attributes and industrial qualifications. This concedes what is commonly called 'equality of opportunity,' repudiates the sex-prejudice by which women workers have been so unjustly handicapped, and, at the same time, discountenances the extravagant claims of certain sections of women that all kinds of artificial grades should be introduced into industry merely to assist the entrance of women. Secondly, the work at which, and the conditions under which, they are employed must be compatible physiologically and psychologically with their sex peculiarities. It cannot and must not be overlooked that women are 'the mothers of the race.' Thirdly, women must not be allowed to undercut and displace men. This is a practical danger which, if it became habitual, would be fatal to industrial concord. Without any doubt, as things are to-day, a woman of efficiency equal to a man, if obtainable—as she is in many cases—can always be secured, especially in unorganised trades, for substantially less remuneration than the man. It is imperative that this should not take place."

### The Lesson.

We make no excuse for giving this short *epitome* of an economic paper. Physiological principles will more and more govern the handling of economic problems in the future. It is to the medical profession that the Government, the employer, and the Labour leader will turn for advice on these principles. We must therefore study to a greater degree than we have done in the past each economic problem as it comes to the fore. Perhaps at the same time we should remember that we must continue to study physiology long after the time that it has been customary to do so, in order that the advice we give may be based upon modern and up-to-date knowledge.

